# Transversus Abdominis Plane (TAP) Block: A Comparative Study between Levobupivacaine versus Levobupivacaine plus Ketamine in Abdominoplasty

**DOI:** 10.1155/2021/1762853

**Published:** 2021-10-31

**Authors:** Radwa F. Mansour, Mohamed A. Afifi, Mohamed S. Abdelghany

**Affiliations:** ^1^Lecturer of Anesthesia and Surgical ICU, Faculty of Medicine, Tanta University, Tanta, Egypt; ^2^Lecturer of Anesthesia and ICU, Alazhar Faculty of Medicine in Cairo, Alazhar University, Cairo, Egypt

## Abstract

**Purpose:**

We conducted this study to explore the hypothesis that the addition of ketamine to levobupivacaine in ultrasound-guided Transversus Abdominis Plane (TAP) block would result in a better and prolonged duration of postoperative analgesia for patients undergoing abdominoplasty. *Material and Methods*. This randomized prospective study was conducted on 50 patients who were scheduled for abdominoplasty. TAP block was performed bilaterally for all patients either with levobupivacaine 0.5% 15 ml plus ketamine 0.5 mg/kg in a total volume of 20 ml in the LK group (*n* = 25) or with levobupivacaine 0.5% 15 ml plus 5 ml normal saline in a total volume of 20 ml in the L group on each side.

**Results:**

Visual analogue scale (VAS) was significantly lower in the LK group in resting condition at 6, 12, and 16 h postoperatively compared to the L group. On movement, the VAS was significantly lower at 4, 6, 8, 12, 16, and 24 h postoperatively in the LK group compared to the L group. The time for first rescue analgesia was longer in the LK group (18.7 ± 4.8 h) than that in the L group (6.5 ± 2.4 h) with the reduced total amount of rescue morphine in the LK group (1.14 ± 2.2 mg) versus the L group (5.86 ± 3.6 mg). Only six patients in the LK group requested rescue morphine, whereas nineteen patients requested rescue morphine in the L group.

**Conclusions:**

In TAP block, adding ketamine 0.5 mg/kg enhanced the analgesic efficacy of levobupivacaine 0.5% in patients undergoing abdominoplasty and reduced the required analgesics postoperatively.

## 1. Introduction

One of the most popular aesthetic surgeries is abdominoplasty, which aims to remove excess skin and fat from the abdomen with or without plication of the rectus sheath and liposuction [1, 2]. Postoperative pain management has great concern for both the patient and surgeon. Different modalities of analgesia have been employed to decrease pain after abdominoplasty, such as nonsteroidal anti-inflammatory drugs, systemic opioids, epidural analgesia, wound infiltration by local anesthetics, and regional nerve block [3, 4].

Transversus Abdominis Plane (TAP) block is a peripheral nerve block that provides analgesia for the anterior abdominal wall from T6-L1 following abdominal surgery. It was described by Rafi in 2001 [5]. The introduction of ultrasound guidance has allowed the technique to be easy to perform and of increased safety and enhanced the quality through direct visualization [6]. TAP block has been administrated as part of multimodal analgesia for patients scheduled for abdominoplasty to decrease postoperative analgesic requirements [7].

Levobupivacaine is a commonly used local anesthetic (LA), but it has a limited duration of analgesia [8]. This has warranted the addition of adjuncts to enhance the quality and duration of analgesia. Ketamine is an N-methyl-D-aspartate (NMDA) receptor antagonist. It has been used as an adjunct to LAs in peripheral nerve blocks and neuraxial anesthesia [9, 10].

Therefore, this study aimed to explore the hypothesis that the addition of ketamine to levobupivacaine in ultrasound-guided transversus abdominis plane (TAP) block would result in a better and prolonged duration of postoperative analgesia for patients undergoing abdominoplasty.

## 2. Material and Methods

This double-blinded, randomized, and prospective study was conducted at the plastic surgery department after approval of the ethical committee of Faculty of Medicine, Tanta University Hospital (approval number 30915/05/16), followed by registration in the Pan African Clinical Trial Registry (PACTR201703002081320) https://pactr.samrc.ac.za/TrialDisplay.aspx?TrialID=2081. Written informed consent was obtained from 50 patients aged 25–50 years with ASA I and II who were scheduled for abdominoplasty under general anesthesia from March 2017 to August 2019.

Patients with a body mass index “BMI” > 35 kg/m^2^, allergy to drugs used, refused participation, coagulation disorders, alcohol or drug abuse, and mental disorder which interferes with visual analogue scale (VAS) evaluation were excluded from the study.

Randomization was performed using the computer-generated list in a closed sealed envelope to randomly allocate the patients into two groups in a 1 : 1 ratio (25 patients in each group) depending on the drug used. TAP block was performed bilaterally for all patients either with levobupivacaine 0.5% 15 ml plus ketamine 0.5 mg/kg in a total volume of 20 ml in the LK group (levobupivacaine + ketamine) or with levobupivacaine 0.5% 15 ml plus 5 ml normal saline in a total volume of 20 ml in the L group (levobupivacaine only) on each side. The study drugs were prepared by an anesthesiologist not involved in the study. The block was performed by another anesthesiologist experienced in the TAP block.

Preoperative assessment and instructions on how to assess the postoperative pain using VAS from 0 = no pain to 10 = worst pain were conducted to all patients.

General anesthesia and monitoring, including ECG, pulse oximetry, noninvasive blood pressure, and capnography, were standardized for all patients. On arrival at the operating room (OR), midazolam (0.02 mg/kg) was given to all patients. Face mask oxygenation for 5 min was carried out, followed by intubation after induction of anesthesia with propofol (2 mg/kg), cisatracurium (0.15 mg/kg), and fentanyl (1–2 *μ*g/kg). Sevoflurane with oxygen air mixture (50 : 50) was administered for anesthesia maintenance. Controlled mechanical ventilation was adjusting to maintain end-tidal CO_2_ between 35–40 mmHg. All patients received 4 mg of ondansetron. Increments of cisatracurium (0.01 mg/kg) were applied as needed, and fentanyl (1 *μ*g/kg) was given to maintain heart rate and mean arterial blood pressure within 20% of the baseline. Warmer was applied to avoid intraoperative hypothermia. 10 ml/kg/h of Ringer lactate solution was infused throughout the operation.

### 2.1. The Technique of Ultrasound-Guided TAP Block [6]

After induction of anesthesia, TAP block was performed bilaterally with the guidance of ultrasound (Sonoscape SSI-6000, China) and before surgical incision. An anesthesiologist skilled in TAP block stands on the contralateral side of the block after skin sterilization with povidone-iodine 10%. The high-frequency (6–12 MHz) linear transducer was coated with sterilized disposable drape and placed in the midaxillary line between iliac crest below and costal margin above. Advancement of the needle (22 gauge, 100 mm, Stimuplex A, B. Braun, Germany) using in-plane visualization was performed from medial to lateral. After positioning the needle between the internal oblique muscle and the transversus abdominis muscles, negative aspiration was performed before injecting the local anesthetic to avoid any vascular puncture. Injection of 1 ml of normal saline was performed to identify the correct placement of the needle, followed by injection of the blinded study solution in the form of two boluses, one bolus inferolateral (below the umbilicus), and other bolus superolateral (above the umbilicus). On the contralateral side, the same procedure was employed. Following TAP block, abdominoplasty was performed with the same techniques and by the same surgeon for all patients.

At the end of the procedure, sevoflurane was discontinued and reversal of neuromuscular blockade with 0.02 mg/kg atropine and neostigmine 0.05 mg/kg was carried out, followed by tracheal extubation, and then, patients were transferred to the postanesthesia care unit (PACU).

All patients received paracetamol 1 g intravenously repeated every 6 h and ketorolac 30 mg intramuscularly repeated every 12 h as part of the analgesic regimen postoperatively.

Visual analogue score (VAS) was used to evaluate postoperative pain at rest and on movement (knee flexion) at the PACU and then at 2, 4, 6, 8, 12, 16, 20, and 24 h in the patient ward. Morphine 0.05 mg/kg was given as rescue of analgesia when VAS was more than or equal to 4. The number of patients who requested analgesia, time to the first dose of morphine, and the total morphine consumption in the first 24 h were estimated. Postoperative nausea and vomiting were recorded. Any drug- or technique-related complications (psychomimetic changes as hallucination or agitation and local anesthetic toxicity) were documented. Also, sedation score assessed on a 4-point scale (where 0 = alert, 1 = quietly awake, 2 = asleep but easily aroused, and 3 = deep sleep) was recorded. Patients' satisfaction with pain control was evaluated using five-point Likert's score at the end of 24 h. The assessment was conducted by an anesthesiologist who was blinded to group allocation.

VAS was our primary outcome. The secondary outcomes were the time for first analgesic requirements, the total morphine consumption during 24 h, the number of patients who requested analgesia, and patients' satisfaction.

### 2.2. Sample Size

Sample size calculation suggested a minimum of 22 patients in each group based on the results of a previous study [11] to detect a significant difference in VAS at rest of at least 20 mm at *α* error of 0.05, the standard deviation of 23 mm. and power of the study of 80%. We enrolled 25 cases per group to overcome possible dropouts.

### 2.3. Statistical Analysis

Data were fed to the computer and analyzed using IBM SPSS software package version 20.0 (Armonk, NY: IBM Corp). The Kolmogorov–Smirnov test was used to verify the normality of variables distribution; comparisons between groups for categorical variables were assessed using the chi-square test (Fisher or Monte Carlo). Student's *t*-test was used to compare two groups for normally distributed quantitative variables. The Mann–Whitney test was used to compare between two groups for not normally distributed quantitative variables. The significance of the obtained results was judged at the 5% level.

## 3. Results

**Figure 1:**
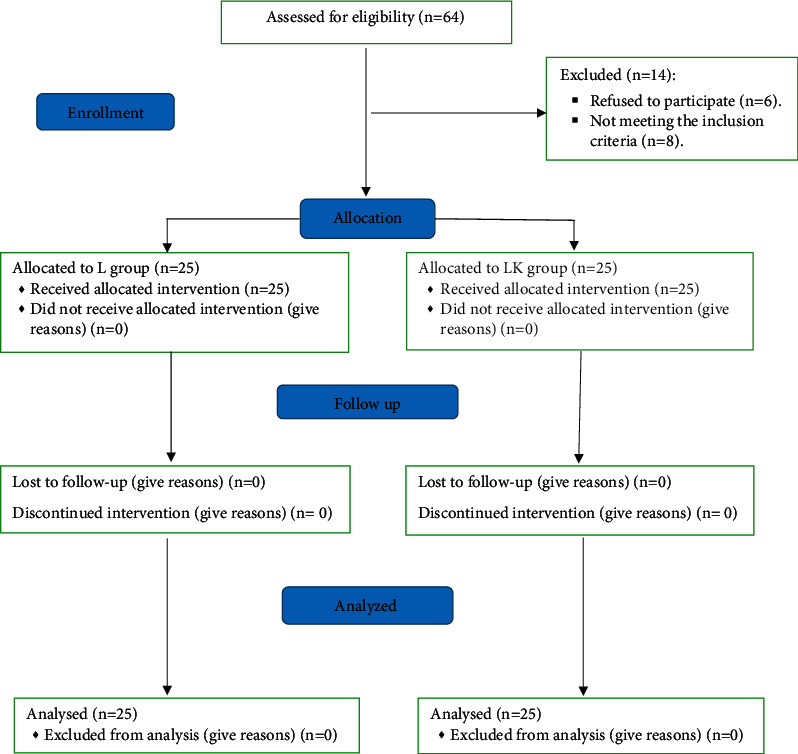
Consort flow chart.

Fifty patients out of eligible 64 patients were enrolled in this study (Figure 1). The demographic data and the duration of the surgery were comparable between both groups (Table 1).

**Figure 2:**
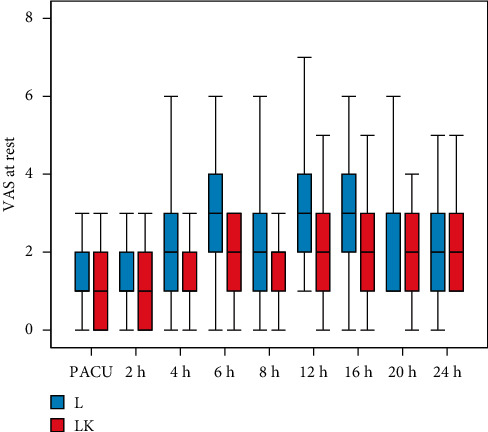
Visual analogue scale (VAS) at rest.

**Figure 3:**
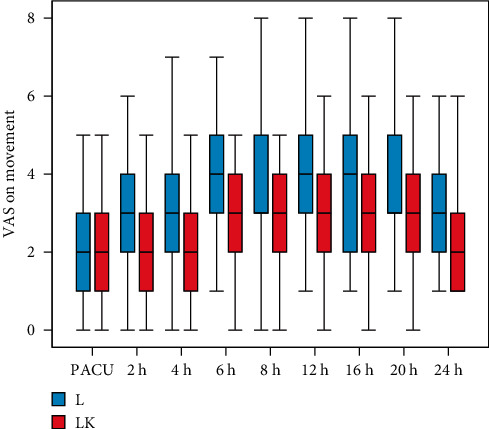
Visual analogue scale (VAS) on movement.

Visual analogue scale (VAS) was significantly lower in the LK group at resting condition at 6, 12, and 16 h postoperatively compared to the L group (Figure 2). On movement, the VAS was significantly lower at 4, 6, 8, 12, 16, and 24 h postoperatively in the LK group compared to the L group (Figure 3).

The time for first rescue analgesia was longer in the LK group (18.7 ± 4.8 h) than that in the L group (6.5 ± 2.4 h). Also, the total amount of rescue morphine was reduced in the LK group (1.14 ± 2.2 mg) versus the L group (5.86 ± 3.6 mg). Only six patients in the LK group requested rescue morphine, whereas nineteen patients requested rescue morphine in the L group (Table 2).

The percentage of patients' satisfaction was higher in the LK group (*P*=0.006). No significant difference regarding side effects (nausea, vomiting, sedation, and psychomimetic changes) was detected between both groups (Table 3).

## 4. Discussion

Abdominoplasty became one of the most commonly performed aesthetic procedures over the past several years. It is one of the most painful procedures in aesthetic surgery due to the large surgical field, extensive dissection of tissues, plication of abdominal wall muscles, and extensive liposuction. Every step of that procedure provides great pain, so reducing postoperative pain and enhancing recovery is quite challenging. TAP block has been established as a trusted technique for postoperative analgesia after abdominal surgeries that allow early mobilization and recovery; therefore, it can be used as part of a multimodal analgesic approach after abdominoplasty. The performance of the TAP block with the guidance of ultrasound enhanced the quality of the block, allowed accurate positioning of local anesthetics in the correct plane, and decreased the incidence of complications [6].

To our knowledge, the literature review did not reveal any study describing the addition of ketamine to levobupivacaine for TAP block in abdominoplasty. Levpubivacaine is an S-isomer of racemic bupivacaine, which is less cardio, neurotoxic, and equally potent to bupivacaine. Recently, multiple glutamate receptors have been found in peripheral nerve terminals and may contribute to peripheral pain signaling. Injection of NMDA receptor antagonists such as ketamine attenuates pain signals [12].

Ketamine blocks central and peripheral NAMD receptors producing antinociceptive effects. Also, ketamine can enhance analgesia through the inhibition of nitric oxide synthase [13]. Another mechanism that can explain ketamine antinociceptive action is sensitization of the opioid system adding to aminergic (noradrenergic and serotonergic) activation with reuptake inhibition. Additionally, ketamine can produce anti-inflammatory effects that decrease the inflammatory response that occurred early postoperatively [14] and also regulate the mechanisms involved in the chronic pain pathology [15].

The major findings of this study are that adding ketamine to levobupivacaine in the TAP block results in significantly lower VAS in resting conditions at 6, 12, and 16 h and on movement at 4, 6, 8, 12, 16, and 24 h postoperatively compared to the levobupivacaine group. Adding ketamine provides prolonged postoperative analgesia up to 18 h versus 6 h in the levobupivacaine-only group and decreased the total amount of rescue morphine (1.14 ± 2.2 versus 5.86 ± 3.6 mg). The number of patients who requested analgesia were higher in the L group (76%) in comparison with the LK group (24%).

Similar to our study, Locatelli et al. reported that perineural administration of ketamine in combination with levobupivacaine enhanced LA blockade and prolonged the postoperative analgesia during caudal anesthesia for lower abdominal and urological surgery [16].

Another study conducted by Othman et al. [9] showed that adding ketamine 1 mg/kg in modified pectoral nerve block to bupivacaine 0.25% in patients who go through modified radical mastectomy prolongs the time to first rescue analgesia with decreased total morphine consumption. Furthermore, El Mourad and Amer [17] evaluated the effects of adding either ketamine 50 mg or dexamethasone 4 mg to bupivacaine 0.5% for the thoracic paravertebral block in breast cancer surgery. They evidenced the beneficial effects of long duration of postoperative analgesia and reduced the total analgesic consumption.

Many studies have found that the addition of ketamine to LA in central neuraxial blocks and peripheral nerve blockades in a human was a safe and effective way to potentiate the LA effect and reduce the required analgesics in the postoperative period [12, 18, 19].

In contrast to our results, adding ketamine 30 mg in interscalene brachial plexus block to bupivacaine 0.5% in a study conducted by Lee et al. [20] showed no enhancement of the onset and the duration of the sensory or the motor blockade. Omar et al. [21] concluded in their study that adding ketamine (0.5 mg/kg) or tramadol (1.5 mg/kg) to bupivacaine 0.5% in paravertebral block dose not enhance the postoperative analgesia.

Ketamine side effects, either cardiovascular or psychomimetic changes, were not observed in any case of the LK group; this could be explained by many ways, e.g., lengthy operation in which the psychomimetic effect of ketamine is masked by the general anesthesia, premedication with midazolam, the use of a low dose of ketamine (0.5 mg/kg), and finally, the relatively slower rate of absorption due to low vascularity in TAP.

One of the limitations to the current study is that we did not estimate the serum concentration of ketamine to evaluate whether the action was related to its local effect or due to systemic absorption. Another limitation is that the TAP block was performed after induction of general anesthesia, so we could not evaluate the success rate of the block. Further studies are needed with different doses of ketamine to determine the ideal one.

## 5. Conclusions

In TAP block, adding ketamine 0.5 mg/kg enhanced the analgesic efficacy of levobupivacaine 0.5% in patients undergoing abdominoplasty and reduced the required analgesics postoperatively.

## Figures and Tables

**Table 1:** Demographic data and the duration of the surgery

	Group L	Group LK	*P* value
Age (y)	35.2 ± 7.4	34 ± 6.1	0.534
Weight (kg)	76.4 ± 5.3	74.4 ± 5.3	0.205
Height (cm)	160.7 ± 3.7	160.4 ± 4.2	0.775
BMI (kg/m^2^)	29.6 ± 2.4	28.9 ± 1.8	0.267
Sex (female)	22 (12%)	23 (92%)	1.000
(male)	3 (88%)	2 (8%)	
ASA I/II	17/8	21/4	0.596
Duration of the surgery (min)	183.6 ± 22.6	190.4 ± 22	0.284

BMI: body mass index. ASA: American Society of Anesthesiologists. L- levobupivacaine, LK- levobupivacaine + ketamine. Data are expressed as mean ± SD (standard deviation) or patient number (percentage (%)). *P* < 0.05 is considered significant.

**Table 2:** Time to the rescue analgesia, the total analgesic consumption, and the number of patients who requested rescue analgesia.

	Group L	Group LK	*P* value
Time to the first rescue analgesia (h)	6.5 ± 2.4	18.7 ± 4.8	<0.001^*∗*^
	6 (4–12)	18 (12–24)	
Total analgesic consumption (mg)	5.86 ± 3.6	1.14 ± 2.2	<0.001^*∗*^
Number of patients who requested rescue analgesia (%)	19 (76%)	6 (24%)	0.001^*∗*^

L- levobupivacaine, LK- levobupivacaine + ketamine. Data are expressed as mean ± SD (standard deviation), median (range), or patient number (percentage (%)). *P* < 0.05 is considered significant.

**Table 3:** Patients' satisfaction and side effects.

		Group L	Group LK	*P* value
Patient satisfaction	1	0 (0%)	0 (0%)	0.006^*∗*^
	2	3 (12%)	0 (0%)	
	3	9 (36%)	2 (8%)	
	4	7 (28%)	7 (28%)	
	5	6 (24%)	16 (64%)	

Side effects	PONV	3 (12%)	4 (16%)	1.000
	Sedation	0	0	—
	Psychomimetic effect	0	0	—

L- levobupivacaine, LK- levobupivacaine + ketamine. PONV: postoperative nausea and vomiting. Data are expressed as patient number (percentage (%)). *P* < 0.05 is considered significant.

## Data Availability

Data will be made available on request.
